# Building a glaucoma interaction network using a text mining approach

**DOI:** 10.1186/s13040-016-0096-2

**Published:** 2016-05-05

**Authors:** Maha Soliman, Olfa Nasraoui, Nigel G. F. Cooper

**Affiliations:** Department of Anatomical Sciences and Neurobiology, University of Louisville, School of Medicine, Louisville, KY USA; Knowledge Discovery & Web Mining Lab, Department of Computer Engineering & Computer Science, University of Louisville, J.B Speed School of Engineering, Louisville, KY USA

**Keywords:** Text mining, Interaction network, Glaucoma, Relation extraction

## Abstract

**Background:**

The volume of biomedical literature and its underlying knowledge base is rapidly expanding, making it beyond the ability of a single human being to read through all the literature. Several automated methods have been developed to help make sense of this dilemma. The present study reports on the results of a text mining approach to extract gene interactions from the data warehouse of published experimental results which are then used to benchmark an interaction network associated with glaucoma. To the best of our knowledge, there is, as yet, no glaucoma interaction network derived solely from text mining approaches. The presence of such a network could provide a useful summative knowledge base to complement other forms of clinical information related to this disease.

**Results:**

A glaucoma corpus was constructed from PubMed Central and a text mining approach was applied to extract genes and their relations from this corpus. The extracted relations between genes were checked using reference interaction databases and classified generally as known or new relations. The extracted genes and relations were then used to construct a glaucoma interaction network. Analysis of the resulting network indicated that it bears the characteristics of a small world interaction network. Our analysis showed the presence of seven glaucoma linked genes that defined the network modularity. A web-based system for browsing and visualizing the extracted glaucoma related interaction networks is made available at http://neurogene.spd.louisville.edu/GlaucomaINViewer/Form1.aspx.

**Conclusions:**

This study has reported the first version of a glaucoma interaction network using a text mining approach. The power of such an approach is in its ability to cover a wide range of glaucoma related studies published over many years. Hence, a bigger picture of the disease can be established. To the best of our knowledge, this is the first glaucoma interaction network to summarize the known literature. The major findings were a set of relations that could not be found in existing interaction databases and that were found to be new, in addition to a smaller subnetwork consisting of interconnected clusters of seven glaucoma genes. Future improvements can be applied towards obtaining a better version of this network.

**Electronic supplementary material:**

The online version of this article (doi:10.1186/s13040-016-0096-2) contains supplementary material, which is available to authorized users.

## Background

Extraction of biological networks, related to specific diseases or conditions from the scientific literature, is an emerging problem which may be solved with the aid of text mining approaches. Biological networks are important features used for modelling, analysis and simulation of biological systems [1], and for the development of hypotheses from data-sets [2–6]. In general, the inference of an interaction network from text can be sub-tasked as: 1) determination of the source of the text to be searched, 2) identification of the entities to be extracted (genes, proteins, metabolites, diseases), and 3) inference of potential relationships between selected entities. Once these subtasks are resolved, the entities and their relationships can be mapped to the nodes and edges of a biological network. A common aspect for subtasks two and three is their amenability to the use of text mining methods for their resolution.

As for the first subtask, the source of text to be mined can be abstracts or full text articles in collections of scientific publications. While the use of abstracts would be more advantageous due to their concise information content [7–9], an increasing number of text mining approaches make use of full text journals [10]. However, in trying to deal with full text publications, there are technical challenges due to the existence of different formats (pdf, HTML) as well as non-uniform substructure across journals. In terms of the second subtask, there are many examples in the literature in which text mining approaches have been used to infer a relationship between biomarker genes and diseases/disorders, including for example, insulin-resistance [11], Alzheimer disease [12], breast cancer [13], prostate cancer [14], and respiratory disease [15]. Therefore, it is possible to develop putative associations between biomarkers and glaucoma with a text mining approach. The third sub-task is to develop a relation extraction (RE) process to reliably infer binary relationships between the entities previously derived from subtask one. Relationships depend on the type of entities we are dealing with. For example, if an entity is a transcription factor, then the textual terms that reflect regulation (up/down-regulate…, etc.) can be sought in the relation extraction process. If an entity is a protein, then textual terms that reflect activation or binding are sought in the relation extraction process [16, 17]. RE can be a closed or an open process. It is closed when there is a set of relations determined a priori such as, (“activate”, “up-regulate”, “express”) and the extractor predicts one of a finite and fixed set of relations. It is open when no relations are specified in advance [18]. For example, an open RE system that runs over the sentence “*HSPA6* is a potential target gene of *FOXC1*”, will list the following binary relation:

$$ \left( HSPA 6,\ \mathrm{is}\ \mathrm{a}\ \mathrm{target}\ \mathrm{gene}, FOXC1\right) $$

On the other hand, if a closed RE is used, this relation will not be extracted unless the relation “target” was included in the set of relations determined a priori. In general, a closed RE is useful when extracting relations from scientific literature, while an open RE is suitable when extracting relations from the web [19].

Text mining services have evolved rapidly to become an important component of inference pipelines. The next generation of text mining approaches have to deal with the construction of complete text mining systems to aid the inference of interactions or associations between bio entities. OntoGene [20], Anni [21], RLISM [22], and CRAB [23] are examples of such next generation systems. In terms of usage, OntoGene is considered the most integrative because it allows the detection of entities and relationships from selected categories of entities, such as proteins, genes, drugs, diseases, and chemicals. On the other hand, Anni has the advantage of introducing an ontology based interface to MEDLINE, and it is capable of retrieving documents for several classes of biomedical concepts. In addition, RLIMS-P and CRAB 2.0 are topic specific approaches. For example, RLIMS-P targets protein phosphorylation and CRAB 2.0 targets cancer risk assessment.

The goal of this study is to initiate the development of a glaucoma interaction network with the aid of text mining the open access scientific literature housed in PubMed Central (PMC). According to the Glaucoma Research Foundation (GRF), glaucoma is the second leading cause of blindness [24]. It is an invisible disease and gradually steals sight without warning. Generally, it cannot be cured, but it can be controlled [25]. Some reported glaucoma interaction networks were based on genome wide association studies (GWAS) [26, 27] while others focused on interaction networks from genome wide expression studies (GWES) [28, 29] but none have yet been based solely on text mining of the vast swath of PMC literature, where all types of glaucoma studies are covered. Such a network is expected to have a wider coverage than prior efforts because it will not be inferred from a particular type of study but rather from all types of studies related to glaucoma.

## Methods

**Fig. 1:**
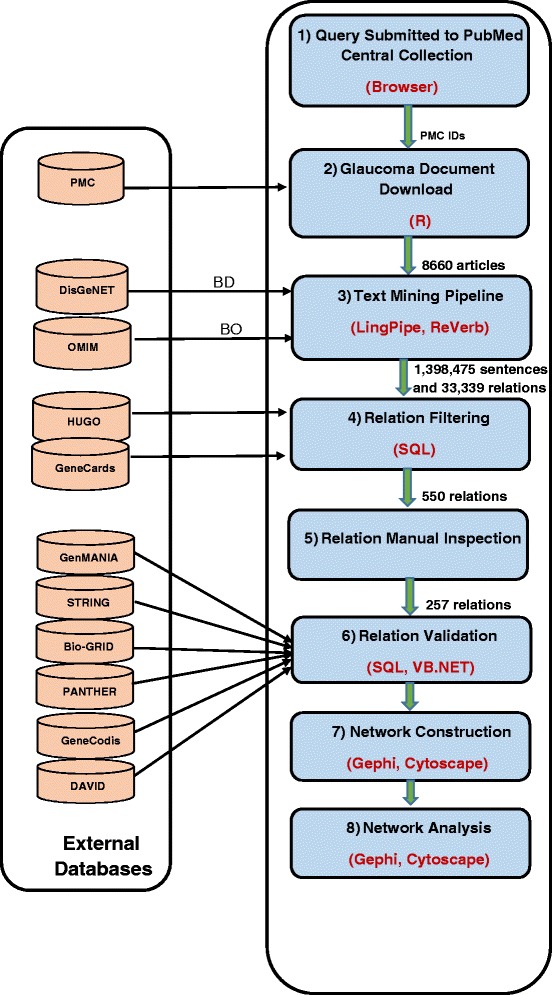
The workflow pipeline followed to build the glaucoma interaction network. Step 1: PubMed Central is queried for glaucoma related articles. Step 2: all glaucoma articles are collected and a glaucoma collection is constructed. Step 3: each document in the resulting collection is processed using the text mining pipeline detailed in Fig. 2 and a set of relations is obtained. Step 4: relations are stored into a database and filtered using SQL queries. Step 5: Filtered relations are subjected to manual inspection to identify meaningful relations worthy of validation. Step 6: inspected relations are then validated and evaluated against external reference databases. Step 7: validated relations are mapped to nodes and edges to form a potential glaucoma network. Step 8: network analysis of the resulting network is performed. The left panel contains external databases needed by each step of the workflow. See Table 1 for definition of BD, and BO

**Fig. 2:**
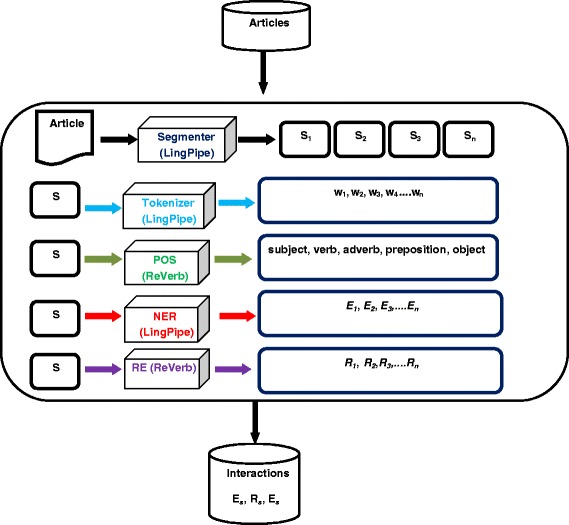
The Text Mining Pipeline. The text mining pipeline that corresponds to step 3 in Fig. 1. First, the segmenter module segments each article into its constituent sentences denoted s_1_ to s_n_. Second, the sentence tokenizer module tokenizes each sentence into a bag of words denoted w_1_ to w_n_. Third, the part of Speech POS module identifies the role of each word in a sentence. Fourth, the name entity recognition module NER extracts gene mentions E_1_, E_2_, E_n_ from the words of the sentence. Finally the relation extraction module (RE) extracts relations R_1_, R_2_, R_n_ from the words of the sentence. The output interaction from applying this sequence of modules is in the form: “Es, Rs, Es” and is saved in a database of interactions

Text mining enables the discovery of useful knowledge from unstructured or semi-structured text [30, 31] which fits the goal of this study. Figure 1 is the flow diagram that shows how the results in this study are generated. The text mining pipeline (Fig. 2), which was used in step 3 of the flow diagram, starts from each article containing some information to be extracted. The article is first segmented into its constituent sentences using a segmenter. Each sentence is then sub-segmented into its constituent words, called tokens, using a tokenizer. Subsequently, part of speech (POS) tagging is applied to each of the tokens to identify the role of each word within the sentence. Additionally, a name entity recognition (NER) is used to identify target entities, which are gene names. Finally, a relation extraction (RE) routine is applied to extract existing relations within each sentence. The relations are then validated, where possible, against an existing reference knowledgebase. Finally, entities and relations are translated into an interaction network. The main tasks in our methodology are:

### Text selection and retrieval

Unlike PubMed, all articles in PubMed Central (PMC) are full text and open access. This makes PMC a suitable repository of the literature for mining full text articles. We used a PubMed medical subject headings (MeSH) terms query to collect all possible glaucoma related articles. PMC Open Access was queried for eight types of key terms related to glaucoma including: “open-angle glaucoma”,”angle-closure glaucoma”,”secondary glaucoma”, “congenital glaucoma”, “hyper glaucoma”, “neovascular glaucoma”, “pigmentary dispersion glaucoma” and”open access”. The resulting data set composed a corpus of 8,660 full length articles ready for mining. Articles were downloaded from PMC Open Access according to the PMC OAI service [32].

### Entity selection and extraction

This study targets the extraction of gene associations which have been previously linked to glaucoma in the open access literature. Our target entities, broadly speaking, are “gene/gene products”. In our approach, we did not make any distinction between mentions of gene, mRNA, or protein in the text. For simplicity, we will reference gene/gene products as “gene”. Association can cover direct protein-protein interaction (PPI) type; predicted or found experimentally, bimolecular events such as expression and localization, and/or static relations. Our definition for association is a loose biological definition that covers any relation that holds between genes or related entities, that is of biological/biomedical or health-related interest, without necessarily implying change [33, 42]. It is for this reason that we have opted for an open RE strategy.

Our glaucoma corpus was segmented into 1,398,475 sentences with the LingPipe sentence segmenter [34]. Genes within sentences were annotated using the LingPipe taggers CharLmHmmChunker and TokenShapeChunker. The performance of any tagger can be evaluated by testing the tagger on an annotated corpus. GenTag [35], and GENIA [36] are well known biomedical annotated corpuses for performance evaluation of taggers. CharLmHmmChunker is trained on GenTag while TokenShapeChunker is trained on GENIA. Compared to GENIA, GenTag is more generic and less specific while GENIA has annotations for 36 biomedical named entities, and therefore provides a breadth classification. Our motivation for using both taggers is to maximize the number of extracted genes [37]. Both taggers accept full length articles as text files and provide an output of annotated files, formatted in Standard Generalized Mark-up Language (SGML) for gene mentions. SGML uses XML tags to describe a mentioned gene but the user will need to specify an encoding system for both input and output files, as well as the desired type of input/output files. For our particular study, we have used the”UTF-8” encoding system, and plain text format for our input/output files.

### Benchmarking genes

A total of 305 glaucoma benchmark genes (BG) were used in this study. Of this number, 155 come from the Online Mendelian Inheritance in the Man database, OMIM® [38] (BO), while the 180 remaining genes come from the Disease Gene Network database DisGeNET release 2.1.0 (July 2014) [39–41] (BD). There were 30 benchmark genes (BC) common to both OMIM and DisGeNET databases (Table 1) indicating their likely importance to glaucoma. The union of OMIM and DisGeNET genes were used as benchmark genes for our intended glaucoma interaction network (Additional File 3). Table 1 lists the benchmark gene types and their abbreviations. Any gene in the literature, which was co-listed in one sentence with one of these BG, is considered a putative association. Sentences, that contain one gene, were filtered out from the tagged sentences to focus our search on sentences that have two or more genes, provided that one of the genes was a BG. If the sentence does not contain a BG, then it is excluded. The idea of the filtering step was to ensure the existence of interacting genes with some BG. The next task is to capture associations between the BG and other non-benchmark genes (NBG), thus constructing a glaucoma interaction network capturing potentially novel relations. The output of this step is a list of associated genes. Some genes were found to be a gene name, a gene synonym, or a previous gene symbol and all of these aliases were mapped to their HUGO approved gene symbol [42].

**Table 1:** Glaucoma benchmark and non-benchmark genes used in building the network

Abbreviation	Definition	Number	Percent
BO	Benchmark glaucoma genes from OMIM database queried with “Glaucoma”	155	51 %
BD	Benchmark glaucoma genes from DisGeNET database queried with “Glaucoma”	180	59 %
BC	Benchmark glaucoma genes from the intersection of OMIM and DisGeNET databases	30 (BO∩BD)	10 %
BG	Benchmark glaucoma genes from union of BO and BD	305 (BO⋃BD)	100 %
NBG	Non-benchmark genes from PubMed Central	150	N/A

For simplicity, benchmark genes used to build the interaction network are abbreviated as BG. If BG are obtained from OMIM, then we call them BO. If BG are obtained from DisGeNET, then we call them BD. Benchmark genes, common to OMIM and DisGeNET, are called BC. Genes that are not benchmark genes are called NBG. The definition, number and percentages of all benchmark genes are listed in columns 2 to 4

### Relation extraction

Sentences that contain putative pairs were subjected to the open source relation extractor ReVerb [43] to extract binary relationships between gene mentions. ReVerb parses each sentence and identifies its main verb. It then starts identifying the subject and object of the sentence. It outputs triplets of “E, Rel, E”, where E is an entity and Rel is a relationship (the main verb of the sentence). In addition to extracted relations, ReVerb also outputs a confidence score associated with the relation that reflects how much ReVerb is certain of its extraction mechanism. Application of ReVerb identified 33,339 binary relations. Extracted relations were verified using the interaction databases GeneMANIA [44] and the Biological General Repository for Interaction Datasets database (BioGRID release 3.4.129) [45]. If the reference databases could not recognize a particular gene in a relation, the gene’s different aliases are first retrieved from GeneCards [46] and the relation is verified using GeneMANIA or BioGRID.

### Network construction

Extracted entities and relations were manually inspected and mapped to nodes and edges. The Gephi open source graph visualization software tool [47] was used to develop a graphic representation of the extracted interaction network (Fig. 7). Analysis of the generated network was carried out with the Cytoscape network analyzer [48]. Enrichment analysis for the extracted genes was conducted through the PANTHER classification system version 10.0 (release May 2015) [49], as well as the Database for Annotation, Visualization and Integrated Discovery (DAVID) [50, 51], and the gene annotations co-occurrence discovery database (GeneCodis) [52–54].

## Results

**Fig. 3:**
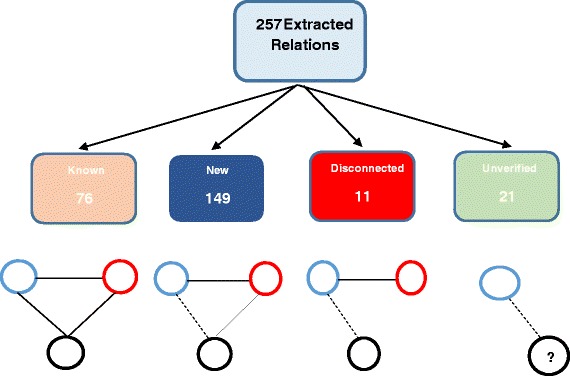
Illustration of the three types of extracted relations found by GeneMANIA in the glaucoma corpus. The total number of extracted relations from the workflow were 257 and they were distributed into 76 known, 149 new, 11 disconnected, and 21 were unverifiable relations. Each type of relation is represented by a picture below it. A known relation is illustrated by three circles directly linked to each other, where a circle represents a gene. A new relation is illustrated by a dotted line between blue and black genes, because an indirect path could be established from the blue to the black gene through the red gene. An unverified relation is illustrated by a question mark in the black gene and a dotted line between the blue and black gene. A disconnected relation is illustrated by the disconnected black gene from the rest of the connected genes

The output from ReVerb may contain incorrect triplets. Therefore, all triplets were saved into a database and were subjected to a filtering process, in which a query is constructed to extract triplets that contained any biological entity name. Filtering ReVerb relations resulted in a total of 550 triplets of “E, Rel, E”, where E is an entity (gene), and Rel is a verb associating the two entities. Some relations from the filtered list of the 550 relations involved “POAG” (Primary Open Angle Glaucoma), while others involve “XFS” (Exfoliation syndrome), a developmental variant of glaucoma (Table 2). The relations included known relations, new relations, disconnected relations, redundant relations, misinterpreted relations, and unverified relations. A known relation is a previously published relation, for example, the relation between *OPTN* and *MYOC*. A relation is defined as new when no direct link between its entities is reported by GeneMANIA or BioGRID. If an indirect link can be established between relation entities through an intervening gene(s), then it is evidence for the possibility of the relation. If no indirect link can be established between relation entities, then it is a disconnected relation, in other words, a relation involving nodes that are currently considered to be disconnected. A redundant relation is a known or new relation, but is repeated many times. A misinterpreted relation is a relation involving an acronym that is identical to a gene symbol, for example *ECD* is an acronym for the endothelial cell density, but was captured as a gene symbol for ecdysoneless homolog gene. An unverified relation is a known or new relation, involving a gene that is not identified by HUGO, GeneCards, or GeneMANIA. Filtering out redundant and misinterpreted relations resulted in a total of 257 unique triplets (E REL E), that include 74 genes from the combined DisGeNet and OMIM databases (BG), 17 of which were common (BC) to both databases (BO, BD), and 150 related genes (NBG) uncovered from the PubMed Central literature database. In terms of the classification of the extracted relations (Fig. 3), 76 were previously known relations, 149 were new relations, 21 were unverified and yet interpretable relations (Table 3) and 11 relations involved disconnected nodes, which linkage could not be confirmed at this time (Table 4) and yet some contextual evidence (Column 5 in Table 4) may suggest some plausible linkage. Both of the 550 and the 257 relations can be found in the Additional files 1 and 2 respectively.

**Table 2:** Genes related to Primary Open Angle Glaucoma (POAG) and Exfoliation syndrome (XFS)

Gene	Disease	Confidence	Support
*MYOC*	POAG	0.98	30
*LOXL1*	XFS	0.98	12
*TG*	XFS	0.98	1
*CYP1B1*	POAG	0.97	12
*GSTT1*	POAG	0.97	4
*CAV1*	POAG	0.97	2
*SPARC*	POAG	0.96	2
*CPE*	POAG	0.96	1
*APOE*	POAG	0.94	7
*CDKN2B-AS1*	POAG	0.94	3
*OPTN*	POAG	0.93	17
*NOS3*	POAG	0.93	5
*WDR36*	POAG	0.92	13
*GLC1A*	POAG	0.92	2
*GLC1N*	POAG	0.92	2
*GSTM1*	POAG	0.91	4
*PDIA5*	POAG	0.89	2
*GC*	XFS	0.88	1
*T*	XFS	0.88	2
*TTR*	POAG	0.87	2
*LOXL1*	POAG	0.86	3
*CDKN2B*	POAG	0.86	2
*SIX1*	POAG	0.85	2
*NTF4*	POAG	0.83	4
*CNTNAP2*	XFS	0.83	1
*GLC3A*	POAG	0.82	2
*OPA1*	POAG	0.81	2
*TBK1*	POAG	0.78	2
*MMP1*	XFS	0.67	1
*MMP3*	XFS	0.67	1
*TP53*	POAG	0.66	1
***ELN***	**XFS**	**0.24**	**1**

The gene and its related disease are listed under the “Gene” and “Disease” columns respectively. The confidence column is the maximum of all confidence values reported by ReVerb for the same relation, extracted from multiple articles. Relations with low confidence are bolded. The support column is the count of articles listing the same gene relation

**Table 3:** Twenty one extracted relations with unverified links from GeneMANIA

Gene1	Gene2	Confidence	Unverified node	PMC Excerpt	PMCID/Year	Remark
*CDKN2B-AS1*	*CDKN2B*	0.93	*CDKN2B-AS1*	CDKN2B-AS1 has been shown to be involved in the regulation of CDKN2B, CDKN2A and ARF expression.	PMC4132588/2014	*CDKN2B-AS1* is a *CDKN2B* antisense. GeneMANIA does not recognize gene anti-sense.
*CDKN2B-AS1*	*CDKN2A*	0.93	*CDKN2B-AS1*	CDKN2B-AS1 has been shown to be involved in the regulation of CDKN2B, CDKN2A and ARF expression.	PMC4132588/2014	CDKN2B-AS1 is CDKN2B antisense. GeneMANIA does not recognize gene anti-sense
*CDKN2B-AS1*	*ARF*	0.93	*CDKN2B-AS1*	CDKN2B-AS1 has been shown to be involved in the regulation of CDKN2B, CDKN2A and ARF expression.	PMC4132588/2014	CDKN2B-AS1 is CDKN2B antisense. GeneMANIA does not recognize gene anti-sense
*CDKN2BAS*	*CDKN2A*	0.92	*CDKN2BAS*	CDKN2BAS also regulates the expression of CDKN2A, a gene previously shown to be down-regulated in other neurodegenerative disorders, including Alzheimer’s disease, suggesting that regulation of CDKN2A expression by CDKN2BAS could also contribute to degeneration of the optic nerve in glaucoma.	PMC3343074/2012	CDKN2BAS is CDKN2B antisense. GeneMANIA does not recognize gene anti-sense
*CNTF*	*LIFRß*	0.90	*LIFRß*	In mouse, human OSM activates the heterodimer of LIF receptor ß (LIFRß and gp130, like CNTF.	PMC4171539/2014	LIFRB is a mouse gene that GeneMANIA did not recognize
*miR410*	*VEGFA*	0.9	*miR410*	Protein levels of VEGFA were also down-regulated with miR410 overexpression and up-regulated with miR-410 interference.	PMC400246/2014	GeneMANIA does not recognize microRNAs.
*STAT1*	*ANRIL*	0.89	*STAT1*	The binding of STAT1 induces the expression of ANRIL, and represses CDKN2B in endothelial cells.	PMC3565320/2013	GeneMANIA does not recognize locus *ANRIL*
*siPITX2*	*DKK1*	0.83	*siPITX2*	DKK1 and KCNJ2 which were shown to be affected by PITX2 siRNAs by real time PCR experiments were each previously reported in one study.	PMC2654047/2009	*siPITX2* is short interfering *PITX2*. GeneMANIA does not recognize short interfering RNAs.
*siPITX2*	*KCNJ2*	0.83	*siPITX2*	DKK1 and KCNJ2 which were shown to be affected by PITX2 siRNAs by real time PCR experiments were each previously reported in one study.	PMC2654047/2009	*siPITX2* is short interfering *PITX2*. GeneMANIA does not recognize short interfering RNAs.
*XCPE1*	*LTBP2*	0.82	*XCPE1*	LTBP2 was predicted to be regulated by KLF4 (at 10 promoters), SP1 (at eight promoters), GATA4 and TEAD (at five promoters) and XCPE1 (at four promoters) was associated with LTBP2.	PMC4019825/2014	*XCPE1* is X gene core promoter element 1 (DNA element). GeneMANIA does not recognize *XCPE1*
*GLC3A*	*GLC3B*	0.78	*GLC3B*	To narrow down the potential candidate CNVs (genes) and match the identified CNVs to target regions and/or genes, we first focused on known chromosomal loci for PCG, namely GLC3A (2p2-p21), which harbors CYP1B1, GLC3B (1p36.2-p36.1), and GLC3C (14q23).	PMC3250374/2011	GeneMANIA does not recognize gene locus
*GLC3A*	*GLC3C*	0.78	*GLC3C*	To narrow down the potential candidate CNVs (genes) and match the identified CNVs to target regions and/or genes, we first focused on known chromosomal loci for PCG, namely GLC3A (2p2-p21), which harbors CYP1B1, GLC3B (1p36.2-p36.1), and GLC3C (14q23).	PMC3250374/2011	GeneMANIA does not recognize gene locus
*E50K*	*TBK1*	0.74	*E50K*	Recently, it was found that E50K mutant strongly interacted with TBK1, which evoked intracellular insolubility of OPTN, leading to improper OPTN transition from the endoplasmic reticulum to the Golgi body.	PMC4077773/2014	GeneMANIA recognizes *OPTN* not its mutated form. *E50K* is a mutation in the *OPTN* gene
*DCDC4*	*PAX6*	0.74	*DCDC4*	The 3′ deletion identified in family 86 contained ELP4 and DCD4, which are located downstream of PAX6.	PMC3044699/2011	*DCD4* (double cortin domain containing 4) is not found in HUGO
*MTMR2*	*NEFL*	0.60	*NEFL*	However, catalytically inactive CMT disease-related MTMR2 mutants lead to NEFL assembly defects and to pathologies similar to the one caused by NEFL mutations, suggesting that MTMR2 and NEFL may function in a common pathway in the development and maintenance of peripheral axons.	PMC3514635/2012	GeneMANIA does not recognize *NEFL.*
*TTRV30M*	*EPO*	0.50	*TTRV30M*	It has been suggested that inhibition of EPO production could be caused by the toxicity of prefibrillar aggregates of TTR V30M.	PMC4087117/2014	GeneMANIA recognizes *TTR* not its mutated form *V30M. V30M* is a point mutation within *TTR*
***BDNF-AS***	***EZH2***	**0.40**	*BDNF-AS*	Further characterization of BDNF-AS indicates that BDNF-AS recruits EZH2 and the PRC2 complex to the BDNF promoter to repress BDNF transcription through H3K27me3 histone modifications.	PMC4047558/2014	*BDNF-AS* is *BDNF* antisense. GeneMANIA does not recognize anti-sense
***BDNF-AS***	***PRC2***	**0.40**	*BDNF-AS*	Further characterization of BDNF-AS indicates that BDNF-AS recruits EZH2 and the PRC2 complex to the BDNF promoter to repress BDNF transcription through H3K27me3 histone modifications.	PMC4047558/2014	*BDNF-AS* is *BDNF* antisense. GeneMANIA does not recognize anti-sense
***BDNF-AS***	***BDNF***	**0.40**	*BDNF-AS*	Further characterization of BDNF-AS indicates that BDNF-AS recruits EZH2 and the PRC2 complex to the BDNF promoter to repress BDNF transcription through H3K27me3 histone modifications.	PMC4047558/2014	*BDNF-AS* is *BDNF* antisense. GeneMANIA does not recognize anti-sense
***siCSTA***	***MYOC***	**0.35**	*siCSTA*	It would be interesting to investigate whether the application of an inhibitor to CSTA, such as its siRNA, could restore the normal MYOC processing and affect the outcome of the disease.	PMC3352898/2012	*siCSTA* is short interfering *CSTA*. GenMANIA does not cover short interfering RNAs.
***Glu50Lys***	***OPTN***	**0.3**	*Glu50Lys*	More, recently, Minegishi and coworkers reported that the over-expression of a glaucoma causing-mutation in OPTN, Glu50Lys, produces an accumulation of insoluble OPTN protein that can be blocked with chemical inhibition of TBK1 activity in HEK293 cells.	PMC4038935/2014	*Glu50Lys* is a mutation in the *OPTN* gene

The genes in each extracted relation are listed under the “Gene1” and the “Gene2” columns respectively. A measure of confidence, reported by ReVerb, is listed under the “Confidence” column, and relations with low confidence (<0.5) are bolded. The unverified node is listed under the “Unverified node” column. The associated text that relates the two genes is listed under the “PMC Excerpt” column. Some genes were identified by their synonyms found in either GeneCards or GeneMANIA. The PMCID of the original article coupled with the year of publication is given under”PMCID/Year” column. Important remarks and gene synonyms may be listed under the “Remark” column

**Table 4:** Eleven extracted relations with disconnected gene nodes from GeneMANIA

Gene1	Gene2	Confidence	Disconnected node	PMC Excerpt	PMCID/Year	Remark
*DCDC1*	*PAX6*	0.96	*DCDC1*	ELP4 and DCDC1 are located downstream of PAX6.	PMC2375324/2008	
*ALB*	*ELP4*	0.93	*ELP4*	ALB was used to normalize ELP4 and PAX6 values for the detection of the relative copy number of the deletion region.	PMC3859656/2013	
*ATOH7*	*FBN1*	0.88	*ATOH7*	We found 10 candidate POAG genes that were highly expressed in both the CPE and NPE (AKAP13, C1QBP, CHSY1, COL8A2, CYP1B1, FBN1, IBTK, MFN2, TMCO1, and TMEM248), three genes that were expressed significantly higher in the CPE (CDH1, CDKN2B, and SIX1), and six genes that were expressed significantly higher in the NPE (ATOH7, CYP1B1, FBN1, MYOC, PAX6, and SIX6).	PMC3909915/2014	
*FBN1*	*TMEM248*	0.88	*TMEM248*	We found 10 candidate POAG genes that were highly expressed in both the CPE and NPE (AKAP13, C1QBP, CHSY1, COL8A2, CYP1B1, FBN1, IBTK, MFN2, TMCO1, and TMEM248), three genes that were expressed significantly higher in the CPE (CDH1, CDKN2B, and SIX1), and six genes that were expressed significantly higher in the NPE (ATOH7, CYP1B1, FBN1, MYOC, PAX6, and SIX6).	PMC3909915/2014	
*GSK3B*	*MTHFR*	0.85	*MTHFR*	For example, GSK3B has a direct connection with IL4 and a secondary connection with MTHFR.	PMC2653647/2009	
*GAPDH*	*VSX1*	0.85	*VSX1*	Each bar represents the relative expression of VSX1 normalized to GAPDH in a different tissue/age; mean ± SD (Sc: sclera, Co: cornea, Ir: iris, CB: ciliary body, Len: lens, Cho:	PMC2267740/2008	
*GLS2*	*HMGB1*	0.80	*GLS2*	the HMGB1 inhibitor GA attenuated diabetes-induced upregulation of HMGB1 and downregulation of BDNF	PMC3671668/2013	*GLS2* is a synonym of *GA*
*SHH*	*ATOH7*	0.78	*ATOH7*	Thus the SHH and GDF11 regulate ATOH7, which in turn regulates Brn3b.	PMC2883590/2010	
***LMX1B***	***COL3A1***	**0.45**	***LMX1B***	Recent immunohistological studies in NPS patients with severe glomerular disease suggest a possible regulation of type III collagen by LMX1B, while the homozygous	PMC2669506/2007	*COL3A1* is a synonym of Type_III_collagen
***NPS***	***PAX6***	**0.05**	***NPS***	Research has demonstrated that retinal neurons and RGCs are mainly comprised of anteriorized NPS that express PAX6 and OTX2.	PMC3747054/2013	
***NPS***	***OTX2***	**0.05**	***NPS***	Research has demonstrated that retinal neurons and RGCs are mainly comprised of anteriorized NPS that express PAX6 and OTX2	PMC3747054/2013	

The genes in each extracted relation are listed under the “Gene1” and the “Gene2” columns, respectively. A measure of confidence, reported by ReVerb, is listed under the “Confidence” column and relations with low confidence (<0.5) are bolded. The disconnected node in the relation is listed under the “Disconnected node” column. The associated text that relates the two genes is listed under the “PMC Excerpt” column. Some genes were identified by their synonyms found in either GeneCards or GeneMANIA. The PMCID of the original article, coupled with the year of publication, is given under ”PMCID/Year” column. Important remarks and gene synonyms may be listed under the “Remark” column

## Analysis and validation

**Fig. 4:**
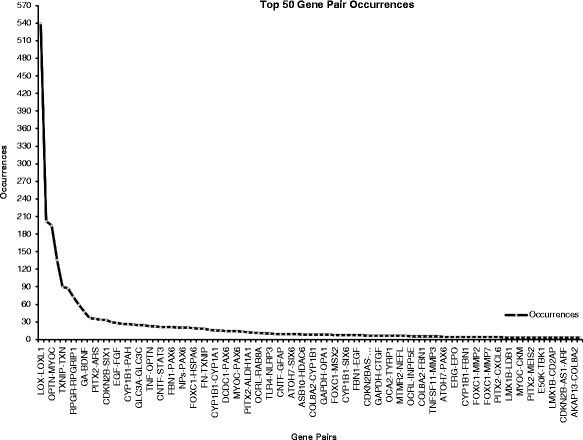
The top 50 gene pair occurrences in our filtered glaucoma corpus. The occurrence frequency of a pair is calculated as the number of articles that has listed this pair in its content. Multiple occurrences of a pair per article is considered one occurrence

The associations between the pair of entities within the 257 extracted triplets (E,Rel,E) were validated against both the GenMANIA database and BioGRID. Validation using BioGRID showed an agreement in only 24 previously known relations with GeneMANIA. Unlike GeneMANIA, BioGRID does not consider the entire gene network for a pair of genes to identify indirect relations as in GeneMANIA. Therefore, all relations, except the 24 known ones, are new according to BioGRID. Most of the 21 unverified relations were due to unrecognized entity symbols in GeneMANIA at the time of writing this paper, such as antisense of a gene (*BDNF*-*AS*, *CDKN2B*-*AS*) or small interfering RNA for a particular gene (*siPITX2*, *siCSTA*), microRNA, general protein family name (M-opsin), and gene variants or mutation (*OPTN* variants: *Glu50Lys* or *E50K*). However contextual evidence (text) from PMC-ID papers (col. 7 in Table 4) suggests some evidence based on the experiments reported in the mined literature. A summary of the different extracted relations and their percentages is listed in Table 5 and the top fifty most frequent relations are depicted in Fig. 4.

**Table 5:** Percentages of extracted relations

Finding Type	Description	Percentage
Known	Verified	76/257 ~ 30 %
New	Can be verified via one or more indirect paths from the known network	149/257 ~ 58 %
Disconnected	Potential discovery that can be verified by lab experiment in the future	11/257 ~ 4 %
Unverified	Gene symbols could not be found in GeneMANIA, HUGO or GeneCards	21/257 ~ 8 %

The Total number of unique and valid relations is 257, which are classified into known, new, disconnected, and unverified relations, respectively. Description and percentage of each class is given under the “Description” and “Percentage” columns

**Fig. 5:**
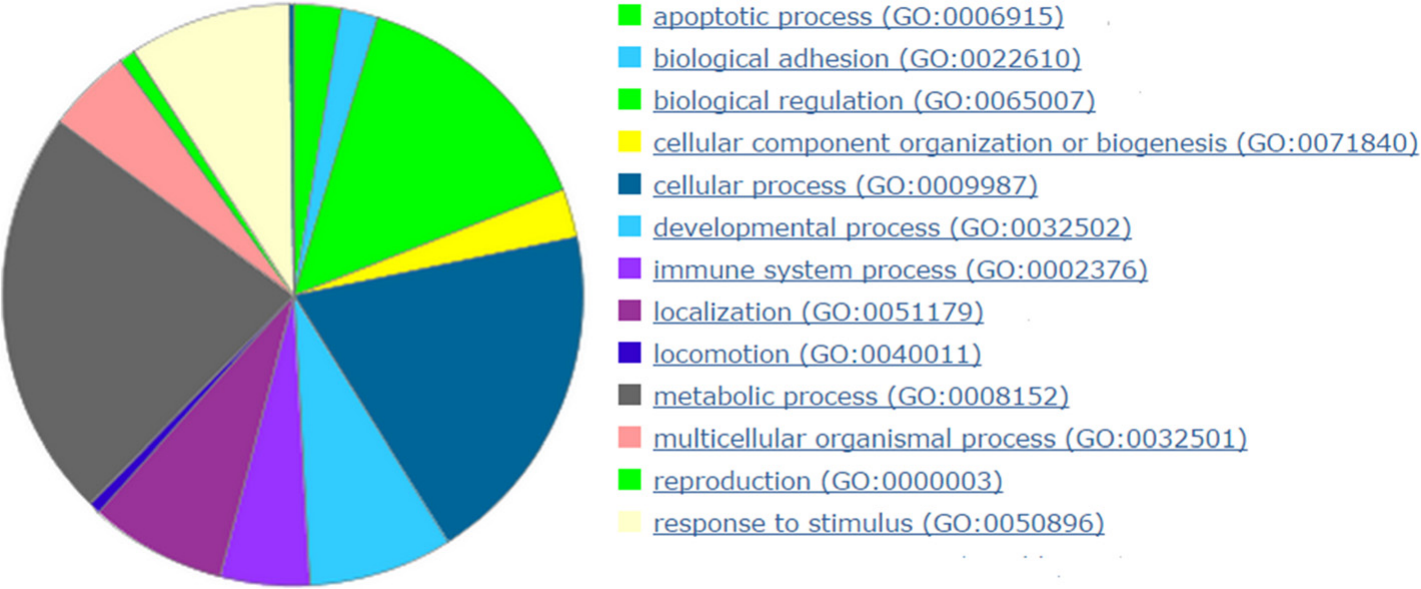
Biological processes associated with extracted non benchmark genes. A pie chart, generated with the aid of PANTHER, with a listing of biological processes associated with 150 extracted non benchmark genes

**Fig. 6:**
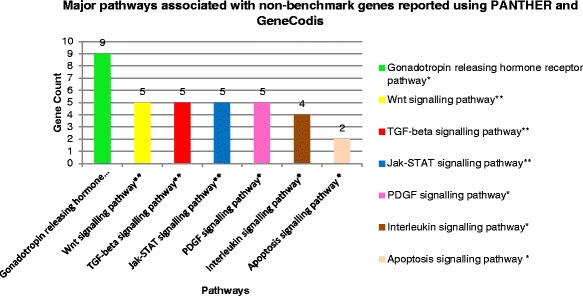
Pathways associated with extracted non benchmark genes. Common pathways reported with the aid of PANTHER and GeneCodis for the 150 extracted non- benchmark genes

As mentioned in the results section, the results included 150 NBG in relation with the 74 BG. The 150 NBG were subjected to enrichment analysis through the PANTHER, DAVID, and GeneCodis databases. We excluded the 74 BG from the functional analysis step to avoid intentionally enriching the results with biological processes and pathways that are already known to be related to glaucoma. PANTHER ranked apoptosis at the top of all biological processes associated with those genes (Fig. 5), which is in line with the evidence that retinal ganglion cell death is a hallmark of glaucoma [55]. The most enriched biological processes, associated false discovery rate (FDRs) and enrichment scores, reported by PANTHER and DAVID clustering, are listed in Table 6. Furthermore, PANTHER identified gonadotropin-releasing hormone receptor (GnRHR) (involving 8.1 % of the total genes on average) and Wnt signalling pathways (involving 4.5 % of the total genes on average) with the highest gene associations. Interestingly, it was recently reported that several Wnt signaling target genes have been identified as potential players in glaucoma pathogenesis [56, 57]. The GnRHR pathway was proposed to control central nervous physiology and pathophysiology modulating cognitive changes associated with aging and age-related neurodegenerative disorders [58]. Combined pathway analysis by PANTHER and GeneCodis is shown with supporting literature (Fig. 6 and Table 7).

**Table 6:** Functional analysis of the 150 extracted non-benchmark genes

Biological Process	Gene Count	Corrected *P*-value
Regulation of apoptosis**	25	2.73E-06
Inflammatory Response*	12	0.002
Immune Response**	17	0.004
Regulation of response to stimulus**	9	0.01
Defense Response*	15	0.01

Biological processes, reported by DAVID, are suffixed by * and are associated with their genes count and corrected *p*-value. Biological processes, that are common to both PANTHER and DAVID are suffixed by ** and are associated with their gene count and corrected *p*-values, obtained from DAVID

**Table 7:** Pathway analysis of the 150 extracted NBG

Pathway name	Count of genes in pathway	FDR	% of genes in pathway	Supporting References
Gonadotropin releasing hormone receptor pathway^b^	9		8.1	[58]
Interleukin signaling pathway^b^	6		5.4	[69]
Wnt signalling pathway^a^	5	0.006	4.2	[56, 57]
Jak-STAT signaling pathway^a^	5	0.001	1.8	[70]
PDGF signaling pathway^b^	5		4.5	[71]
TGF-beta signaling pathway^a^	4	0.01	3.6	[72]
Apoptosis signaling pathway ^b^	2		1.8	[73, 74]

Common pathways, reported by both GeneCodis and PANTHER, are suffixed by ^a^ and the associated false discovery rate (FDR) from GeneCodis is reported. Pathways, reported by PANTHER, are suffixed by ^b^. The percentage of total genes in the pathway is reported along supporting references that link glaucoma to the pathway

Our result is expected to be comprehensive, with partial resemblance to other studies of glaucoma interaction networks. For example, our result shares only 5 and 29 genes with two previous studies [28, 29] respectively. This emphasizes the fact that interaction networks from text mining approaches can be quite comprehensive because they can incorporate and integrate information from all types of studies. Our enrichment analysis also agreed with previously reported enrichments to glaucoma studies [29] such as apoptosis and induction of apoptosis as underlying biological processes and pathways such as *PDGF* signaling pathway, Ras pathway, and apoptosis signaling pathway.

### Network features

**Fig. 7:**
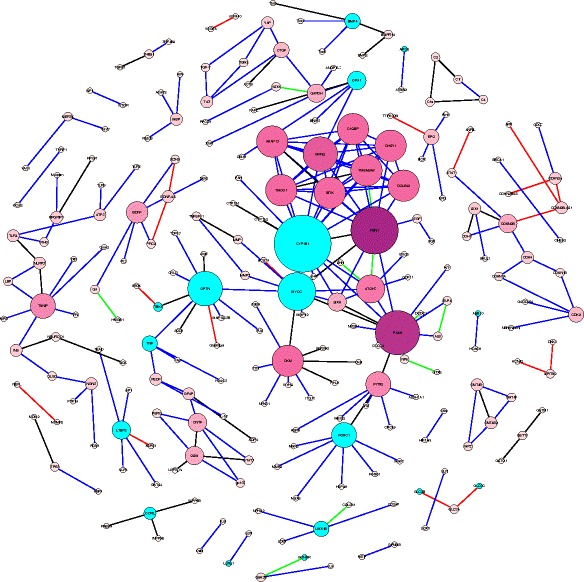
Extracted glaucoma network. Glaucoma network laid with different node sizes. The node size reflects the node degree of a gene where the degree is the total of the number of in-degree and out-degree links. The nodes colored in cyan belong to the BC. The known relations are colored in black. The new extracted relations are colored in blue. The relations with disconnected nodes are colored in green. The relations with unverified nodes are colored in red

**Fig. 8:**
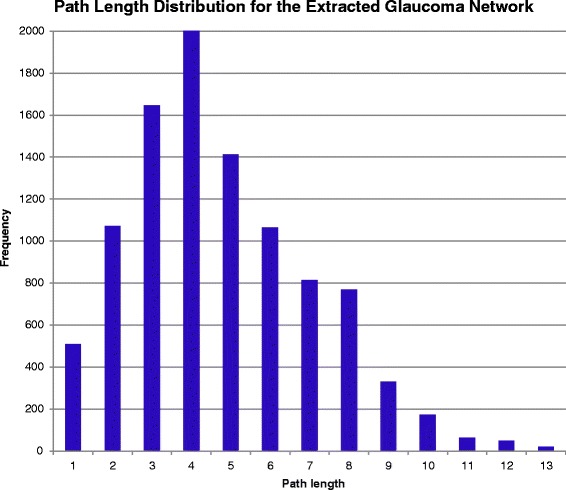
Glaucoma network path distribution by the Cytoscape network analyser

**Fig. 9:**
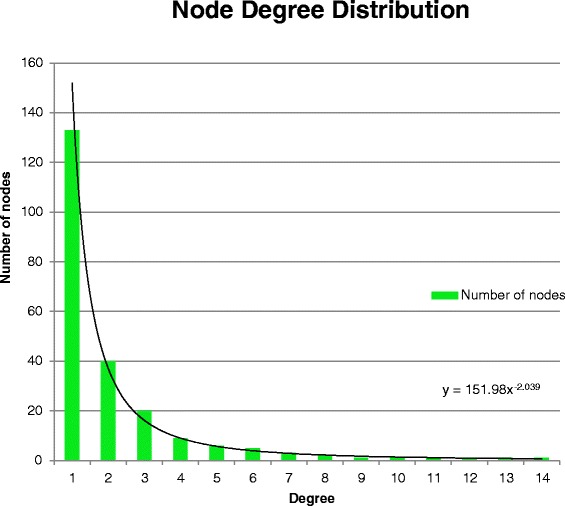
Glaucoma network node degree distribution. The glaucoma node degree distribution, generated by the Cytoscape network analyser, follows a power law fitted to the form *y* = 137.67*x* − ^1.99^

The resulting graph is a scale-free network that follows the Barabási–Albert (BA) network model [59]. A scale-free network is a network with node links that follow a power law distribution, i.e. the probability of linking to a given node is proportional to the number of existing links, *k,* that node has. Our glaucoma network (Fig. 7) consists of 224 nodes and 255 edges. Network analysis shows that the network has a diameter of 13 and a path length distribution as shown in Fig. 8. While the diameter of the network and path length distribution are quantitative measures that offer insight into how well connected a network is, the clustering coefficient describes how clustered the network is. The network diameter is the longest path between all possible pairs of nodes in the network, while the path length distribution summarizes the number of steps along the paths connecting all possible pairs of network nodes. The network has a relatively low clustering coefficient of 0.11; a property which appears to characterize most metabolic networks and protein interaction networks [60, 61], indicating that low degree nodes tend to belong to highly connected neighborhoods, whereas high degree nodes tend to have neighbors that are less connected to each other. The node degree is the number of in-links and out-links for a particular node in the network. The network node degree distribution follows a power law (Fig. 9), another property of scale free networks. Table 8 lists the nodes with top ten degrees, indicating hub entities in the network. To conclude, the current version of the extracted glaucoma interaction network is small but informative. Future versions of the network are expected to evolve closer to a small world network as more links between nodes get added.

**Table 8:** Genes (nodes) with the top 10° in the extracted glaucoma interaction network

Gene(node)	Degree
*CYP1B1*	17
*FBN1*	14
*PAX6*	13
*MYOC*	11
*MFN2*	10
*OPTN*	9
*CKM*	9
*AKAP13*	9
*IBTK*	9
*TMCO1*	9

The degree column represents the total number of a node’s ingoing and outgoing links. Note that *CYP1B1* heads the list with a total of 17 links

### Performance evaluation

As described in the “Methods” section, our text mining pipeline consists of three steps: 1) Text retrieval, 2) Entity extraction, and 3) Relation extraction; each of which has a different associated level of performance. Text retrieval performance is evaluated based on the retrieval of relevant documents. Entity recognition performance is evaluated by the fact that most, if not all genes, should be captured from the collection of glaucoma documents. Relation extraction performance is validated by the extraction of relevant relations. Performance evaluation is usually based on precision (P), recall (R) and F1-score metrics. P is defined as the proportion of retrieved instances that are relevant, while R is the proportion of relevant instances that were retrieved. F1-score combines recall and precision. These metrics are given in Eq (1):

$$ P=\frac{\#\kern0.5em  of\kern0.5em  relevant\kern0.5em  retrieved\kern0.5em  instances}{\#\kern0.5em  of\kern0.5em  retrieved\kern0.5em  instances},\kern0.5em R=\frac{\#\kern0.5em  of\kern0.5em  relevant\kern0.5em  retrieved\kern0.5em  instances}{\#\kern0.5em  of\kern0.5em  relevant\kern0.5em  instances},\kern0.5em F1=\frac{2*P*R}{P+R}(1) $$

The text retrieval step performance metrics and values are listed in Table 9 and Table 10. For the entity extraction step performance, the GENIA tagger targets a broader domain. Hence, it can be expected to tag varied entities (including localization, cell type, DNA, etc.), but possibly less genes/proteins than the GenTag tagger. This is because the latter is more focused towards genes and proteins. Indeed, in our particular study, GENIA tagger tagged 2410 genes while GenTag tagged 3422 genes. Table 11 lists the performance measures, reported in [62] for GENIA and the average performance measures, reported in [63] and [64] for GenTag.

**Table 9:** Distribution of articles in the text retrieval step, depending on their accessibility and relevance

	Relevant	Not Relevant	Total
Retrieved open access articles	7425	1235	8660
Restricted access (not Retrieved)	22733	unknown	__
Total	31393	__	__

Relevant articles are those that contain at least one occurrence of the word “glaucoma” in their text. The portion of restricted access articles, are not relevant, is unknown to us at the time of writing this article

**Table 10:** Evaluation metrics for the retrieval step

Metric	Value
Precision	7425/8660 = 85 %
Recall	7425/31393 = 23 %
F1	36 %

Evaluation metrics are computed based on Table 9. Note that recall is limited by the number of open access articles at this time

**Table 11:** Performance measures of the used LingPipe NER tagger

Tagger	Entity Type	Recall (%)	Precision (%)	F-score (%)
GENIA	Protein	81.41	65.82	72.79
	DNA	66.76	65.64	66.2
	RNA	68.64	60.45	64.29
	Cell Line	59.6	56.12	57.81
	Cell Type	70.54	78.51	74.31
	Overall	75.78	67.45	71.37
GENTAG	Gene/Protein	79	88	70.8

Reported measures for the GENIA tagger is based on the GENIA performance web site [62] while performance measures of the GENTAG tagger is the average of the measures reported in [63, 64]

Because the relation extraction step depends on ReVerb, we report ReVerb’s performance from [43], which were 65 % precision and 52 % recall. Therefore, the F1 score associated with the relation extraction step is estimated at 58 %.

## Discussion

**Fig. 10:**
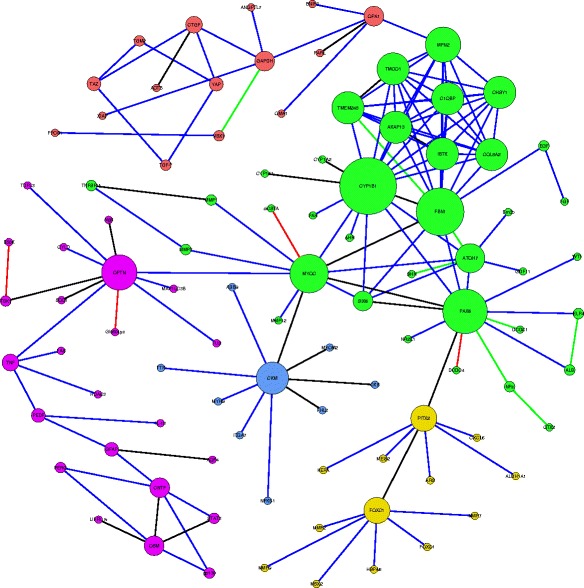
A smaller glaucoma interconnected subnetwork resulting from applying the modularity algorithm in Gephi on the original glaucoma network. The glaucoma network in Fig. 7 was subjected to the Gephi modularity clustering algorithm to identify communities and classes within the network. Five distinct classes colored in green, purple, red, yellow, and blue respectively, can be seen

**Fig. 11:**
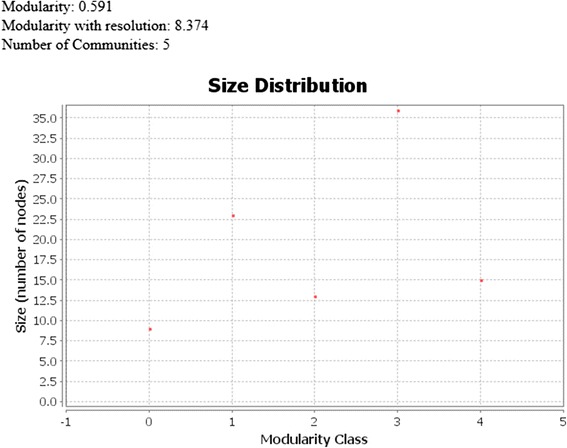
Modularity community classes and associated node sizes. The modularity classes are listed on the X axis while the number of nodes is on the Y axis. The highest number of nodes is 36 in modularity class 3, while the least is 9 in modularity cluster 0. The value of modularity, before and after applying the resolution, is listed on the top left of the figure. A resolution value of 9.0, was used in association with the modularity algorithm to obtain dense, well separated classes

While we have described an expansion of the known network of glaucoma related genes, we were surprised that less than a quarter of the genes extracted from DisGeNet and OMIM combined were connected to our network at this time (74/305 = 24 % BG). Community detection with the Gephi’s Louvain modularity maximization algorithm [65], partitioned the network into five distinct modular clusters (Fig. 10). The Louvain modularity maximization algorithm measures the density of links, inside clusters as compared to links between clusters and uses a resolution measure [66] that measures the flows of probabilities in the network. The resulting five clusters formed a strongly connected subnetwork that is 41 % of the size of the original network (96 nodes and 148 edges), with only the giant influential components (nodes with high connectivity) of the network. Examination of the clusters, showed that each has one or more of the BC genes, making a total of 7 BC. Almost the same ratio is observed with the clusters, where less than a quarter of the 30, genes present in both of OMIM and DisGeNET databases (7/30 = 23 %), are connected to the clusters. As to the BC genes, the green cluster has *CYP1B1* and *MYOC*, the purple cluster has *OPTN*, *TBK1*, and *TNF*, the red, yellow, and blue clusters have *OPA1*, *FOXC1*, and *CMK* respectively. Their representation here supports the notion that the 30 BC are most highly ranked among all of the BG. Table 12 profiles the different properties of each of the five clusters and Fig. 11 depicts the clusters and their sizes.

**Table 12:** Clusters extracted from the giant components in the glaucoma network and their associated profiles

Cluster	# Nodes	BG	NBG	Node with highest degree	Known relations	New relations	Unverified relations	Disconnected relations
Green	36	6	11	*CYP1B1* = 17	10	14	8	4
Purple	23	1	9	*OPTN* = 10	7	15	0	1
Red	15	0	5	*OPA1* = 5	2	12	0	1
Yellow	13	2	5	*FOXC4* = 7	2	11	0	0
Blue	9	2	7	*CKM =* 9	4	5	0	0

The giant components in the glaucoma network depicted in Fig. 7 are clustered into five clusters. Clusters are ordered in descending order of the number of nodes in each cluster. Cluster properties include number of BG, NBG, highest degree, and the number of different types of relations contained within the cluster

The text mining approach, adopted in this study, relies heavily on natural language processing (NLP) methods. We reported in this study, the first version of a glaucoma interaction network, with the intention to report future refined versions when improvements in the text mining pipeline become available. For example, more specificity could likely be added to the results if a better tailored tagger was used. We relied on taggers that were trained on general biological texts that are not specific to glaucoma. Therefore, it is expected that not all entities will be captured from our article collection and an in-house developed tagger, that is trained on literature related to eye diseases and disorders, would likely improve our outcome. Additionally, we note that the currently available glaucoma corpus has a relatively small size compared to other corpora associated with other diseases such as prostate cancer or breast cancer. Since the number of extracted relations is proportional to the size of the corpus, it is desirable to increase the corpus size to discover more relations. There are many possibilities to increase the size of the available glaucoma corpus. For example, PubMed abstracts could be added to the current corpus, or only PubMed abstracts could be considered instead of PMC full text articles. Both options may significantly impact our future results.

Perhaps, the most sought improvement after enlarging the body of literature, would be to reconsider the relation extraction step. ReVerb is designed for open relation extraction, and has not been tweaked for closed relation extraction. In closed relation extraction, the target includes verbs that are known a priori. However, considering our small corpus, it would have negatively affected our extracted relations if we had been confined to a closed set of predetermined verbs [67]. Another difficulty faced by ReVerb is handling complex sentence structures. Although many authors tend to use simple sentence structure such as: Subject-verb-Object, in describing a relationship between two genes, it is not rare for authors to use more complex sentence structures such as conjunctive structure sentences. The latter are sentences that bear multiple verb based relationships or a single verb, to describe many-to-one or one-to-many relationships in a single sentence, respectively. Due to its shallow syntactic analysis, ReVerb’s maximum recall is limited and therefore, it misses most of the conjunctive structure sentences. A better but probably time consuming alternative, is to use an NLP parser such as the Stanford parser [68] to parse target sentences, then search the parsing tree to capture all missing models of verbs.

## Conclusions

In this study, we have constructed a glaucoma interaction network using a text mining approach applied to open access PMC based literature. Our findings revealed 149 potential new relations. These newly discovered relationships link 74 benchmark genes (BG) present in the 2 databases, DisGeNet and OMIM, with 150 non-benchmark genes (NBG) present in the PubMed Central database, in the form of a small world interaction network. These findings include 21 unverified relations and 11 disconnected relations, which could be verified in the lab. The constructed network contains five distinct gene clusters in association with 7 BC. The 5 clusters are interconnected through 4 gene-gene associations which include: *OPA1-MFN2*, *PITX2-PAX6*, *MYOC-CKM* and *MYOC-OPTN*. Thus the larger network is only possible because of these 4 bridges. It is important to note that 2 of these 4 gene-gene bridges, *OPA1-MFN2* and *MYOC-OPTN*, were discovered through this text mining approach which has associated genes in the DisGeNet and OMIM databases with the PubMed Central database. Finally, we have discussed several important issues with text mining approaches which could aid future iterations of disease-based gene-interaction networks.

## Additional files

Additional file 1: Filtered Extracted Relations. (XLSX 58 kb) Additional file 2: Unique Extracted Relations. (XLSX 32 kb) Additional file 3: Glaucoma Benchmark Genes (XLSX 38 kb)
